# Adsorption Technology for PFAS Removal in Water: Comparison between Novel Carbonaceous Materials

**DOI:** 10.3390/ma17174169

**Published:** 2024-08-23

**Authors:** Marco Petrangeli Papini, Marta Senofonte, Riccardo Antonino Cuzzola, Rania Remmani, Ida Pettiti, Carmela Riccardi, Giulia Simonetti

**Affiliations:** 1 Department of Chemistry, University of Rome “La Sapienza”, 00185 Rome, Italy; marco.petrangelipapini@uniroma1.it (M.P.P.); cuzzola.17521513@studenti.uniroma1.it (R.A.C.); rania.remmani@univ-biskra.dz (R.R.); ida.pettiti@uniroma1.it (I.P.); giulia.simonetti@uniroma1.it (G.S.); 2 Department of Technological Innovations and Safety of Plants, Products and Anthropic Settlements, National Institute for Insurance against Accidents at Work (INAIL), 00144 Rome, Italy; ca.riccardi@inail.it

**Keywords:** PFASs, water treatments, adsorption technology, pinewood biochar, date seeds biochar

## Abstract

PFASs are a variety of ecologically persistent compounds of anthropogenic origin loosely included in many industrial products. In these, the carbon chain can be fully (perfluoroalkyl substances) or partially (polyfluoroalkyl substances) fluorinated. Their ubiquitous presence in many environmental compartments over the years and their long-lasting nature have given rise to concerns about the possible adverse effects of PFASs on ecosystems and human health. Among a number of remediation technologies, adsorption has been demonstrated to be a manageable and cost-effective method for the removal of PFASs in aqueous media. This study tested two novel and eco-friendly adsorbents (pinewood and date seeds biochar) on six different PFASs (PFOS, GenX, PFHxA, PFOA, PFDA, and PFTeDA). Batch sorption tests (24 h) were carried out to evaluate the removal efficiency of each PFAS substance in relation to the two biochars. All samples of liquid phase were analyzed by a developed and then a well-established method: (i) pre-treatment (centrifugation and filtration) and (ii) determination by high-performance liquid chromatography coupled with mass spectrometry (HPLC-MS/MS). The results evidenced a comparable adsorption capacity in both materials but greater in the long-chain PFASs. Such findings may lead to a promising path towards the use of waste-origin materials in the PFAS remediation field.

## 1. Introduction

Perfluoroalkyl and polyfluoroalkyl substances (PFASs) are anthropogenic persistent organic molecules characterized by a hydrophobic tail of variable length, in which hydrogen atoms are entirely or partially replaced by fluorine atoms and a polar head with differing functional groups. Because of the strong carbon–fluorine bond, PFASs have excellent thermal–chemical stability and resistance to degradation, which makes them crucial in many industrial applications (e.g., food packaging, textiles, personal care products, fire-retardants, electronics, and pesticides) [1]. PFASs can be divided into two main families in terms of chain length and functional group: perfluoroalkyl sulfonic acids (CnF_2n_+1SO_3_H PFSAs) and perfluoroalkyl carboxylic acids (C_n_F_2n_+1COOH PFCAs). Long-chain carboxylic and sulfonic acids (number of carbons: ≥6), such as PFOA and PFOS, have shown high bioaccumulation properties and have been classified as PBT (Persistent Bioaccumulative Toxic) substances according to the EU REACH (Registration Evaluation and Authorization of CHemicals) [2]. Several biomonitoring trials, carried out to evaluate PFAS health effects on the human population, have highlighted their characteristics of being endocrine disruptors and their potential adverse effects, such as altered metabolism, reduced fertility along with fetal growth, obesity, carcinogenicity, and the reduced ability of the immune system to fight infections [3,4,5,6,7,8,9]. Therefore, many regulations and restrictions have been introduced in both the USA and Europe, resulting in the replacement of proven toxic compounds with some short-chain PFASs (e.g., PFOA to GenX) [10,11,12,13]. Despite this, due to their persistence and bioaccumulation characteristics, PFASs are significantly widespread in all environmental matrices. Surface water contamination is of particular concern since it can easily reach groundwater, making drinking water and process water the most impactful way of human exposure [14]. Thus, the US Environmental Protection Agency (EPA) has set a 10–90 ng L^−1^ threshold value for drinking water, and in Europe, the most recent action plan has established a limit of 0.5 µg L^−1^ for all PFASs in drinking water [2,15,16]. In this context, the development of efficient and cost-effective water treatment methods is a primary challenge for the scientific community. The most effective water treatments for PFAS removal include the use of advanced oxidation, reverse osmosis, ion-exchange resins, and nanofiltration [17]. Even though these technologies have shown promising results, they present concerning drawbacks, such as the formation of shorter-chain PFASs as byproducts. Moreover, the low concentration of PFASs in water and their high hydrophilicity make it difficult to provide an overall efficient remediation [17,18,19]. Among the most effective treatment processes mentioned, adsorption is an established technology for the removal of contaminants, both as a stand-alone application and in combined water treatment plants [17]. For example, activated carbon (AC) has been successfully used due to its highly specific area and porosity [20,21]. However, some disadvantages, such as cost regeneration and the resulting reduced adsorption efficiency, have led the scientific community to test novel materials capable of overcoming these limitations. Over the years, the eco-friendlier biochar material has been taking hold as a cost-effective remediation treatment for organic compounds: its major advantage lies in being a carbon-rich waste material derived from the slow pyrolysis of biomass that does not require activation by solvents or gas. In addition, its 3D structure enables the material to easily interact with a variety of substances through its hydrophobic surface, making biochar a potentially suitable material also for PFASs [22,23,24]. Moreover, from a circular economy perspective, the demand for materials deriving from food and manufacturing waste is steadily increasing. Hence, the aim of the present study was to test two novel and eco-friendly biochars for the removal of PFASs in aqueous media. The two chosen adsorbents, made from pinewood and dates, have been successfully used in the remediation of organic compounds [25]. PFASs were selected to investigate—as much as possible—a wide spectrum of chemical structures by different chain lengths (6–14 carbons) and functional groups (carboxyl and sulphonic).

## 2. Materials and Methods

### 2.1. Chemicals and Materials

Six PFAS standards of different chain lengths (i.e., C_4_–C_14_) and compositions were investigated: perfluorohexanoic acid (PFHxA), ammonium perfluoro (2-methyl-3-oxahexanoate) (GenX), perluorooctanoic acid (PFOA), perfluoroctanesulfonic acid (PFOS), perfluorodecanoic acid (PFDA), and perfluorotetradecanoic acid (PFTeDA) (Table S1). Two mass-labeled internal standards (ISs) (i.e., ^13^C_4_-PFHxA and ^13^C_4_-PFOS) (LGC standard Ltd., Milano, Italy) were also used. The adsorbent materials were received thanks to a collaboration between the Burkhardt GmbH company and the University of Briska: the two biochars were produced from pinewood (PW) gasification in the form of powder material (Burkhardt GmbH, Mühlhausen, Germany) [26] and the high-temperature pyrolysis of date seeds (DSs) (Briska University, Algeri, Algeria), respectively.

### 2.2. Batch Adsorption Tests

**Figure 1:**
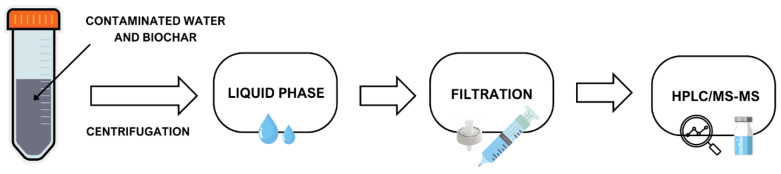
Laboratory procedure from batch test to analytical determination.

The experiments were performed in two steps: (i) adsorption kinetic tests to establish the equilibrium time and (ii) adsorption isotherms tests to investigate the removal capacity of both selected biochars. In detail, for kinetics tests, an aliquot of the solution spiked with a PFAS compound was sampled at the beginning of the test (time 0) to evaluate the starting PFAS concentration (C_0_) and C_t_ after 1, 2, 3, 4, 5, 6, 7, 8, 24, 27, 30, 54, and 57 h. The equilibrium concentration was achieved after 24 h (C_t_ = 24 h): four additional samplings at different time intervals were also carried out after 24 h to confirm the achievement of equilibrium. A solid/liquid ratio of 2 g L^−1^ was chosen for the experiments and a fixed quantity of sorbent material (0.002 g) was placed in contact with contaminated solutions at different concentrations (0.5, 1, 2.5, 5, 6, 8, 10, 20, 30, and 40 mg L^−1^). The equilibrium concentration (C_t_) determined was used to calculate the sorption equilibrium amount (q_t_) at that time with the following Equation (1):

(1) $$q_{t}=\frac{\left(C_{0}-C_{t}\right)V}{m}$$

where C_0_ (µg L^−1^) is the initial concentration of the single PFAS in the solution and C_t_ (µg L^−1^) is the concentration of the PFAS in the solution at the time of collection t (h); V (L) is the volume of the solution; and m (mg) is the weight of the DS and PW biochar. Batch isotherm experiments were set one compound at a time for 24 h in order to reach the equilibrium and carried out on an orbital shaker at 180 rpm, and the biochar of choice was added at different quantities (from 30 to 250 mg) to 50 mL of ultra-pure deionized water (MilliQ) spiked with a PFAS standard solution at a fixed concentration (10 mg L^−1^; 1–5%RSD) in a 50 mL polypropylene tube (Falcon). The samples collected were then centrifugated at 4200× *g* rpm for about 20 min and then 3 mL of each sample was filtered with a 0.22 µm of cellulose acetate filter (Sartorius Stedim). Lastly, an internal standard (IS) for each sample was added for the instrumental analysis (Figure 1). The equilibrium concentration (C_e_) determined was used to calculate the sorption equilibrium amount (q_e_) with the following Equation (2):

(2) $$q_{e}=\frac{\left(C_{0}-C_{e}\right)V}{m}$$

where C_0_ is the starting PFAS concentration expressed in mg L^−1^; q_e_ is the PFAS sorption amount in mg g^−1^; C_e_ is the equilibrium PFAS concentration expressed in mg L^−1^; V is the volume of the solution in L; and m is the amount of sorbent in mg.

### 2.3. Equilibrium and Kinetic Modeling

The adsorption kinetic model was derived from the *Lagergren* pseudo-first-order equation as reported in Equation (3):

(3) $$\frac{dq_{t}}{dt}=k\left(q_{e}-q_{t}\right)$$

where q_t_ and q_e_ are the amount adsorbed at time t and at equilibrium and k_1_ is the rate constant of the pseudo-first-order sorption process. The integral equation after applying the initial conditions of q_t_ = 0 at t = 0 is Equations (4) and (5):

(4) $$ln\left(q_{e}-q_{t}\right)=lnq_{e}-kt$$

or

(5) $$q_{t}=q_{e}(1-e^{-kt})$$

Equilibrium tests were carried out to investigate both DS and PW sorption capacities and their affinity for the target PFAS compounds. Two isotherm models were attempted for the construction of the equilibrium curves: the Langmuir and the Freundlich models. The models were both applied to each experimental plot in deionized water. The Langmuir and Freundlich models are reported in Equations (6) and (7), respectively:

(6) $$q_{e}=q_{max}\frac{K_{L}C_{e}}{{1+K}_{L}C_{e}}$$

(7) $$q_{e}=K_{F}{C_{e}}^{n}$$

where q_max_ (mg g^−1^) is the maximum adsorbable amount; K_L_ is the Langmuir thermodynamic constant (L mg^−1^); K_F_ is the Freundlich (L mg^−1^); and n is a dimensionless parameter greater than zero—n > 1 means upwards concavity, whereas n < 1 represents downward concavity.

### 2.4. HPLC/MS-MS

The analytical determination performed in this study was modified and adapted from a previous study [27] for the PFAS water sample analysis (Tables S2 and S3). Briefly, every sample was analyzed using a coupled system consisting of a high-pressure liquid chromatography Agilent 1290 (HPLC) and an Agilent G6460 triple-quadrupole mass spectrometer (QqQ MS/MS) (Agilent Technologies, Toronto, CA, Canada), with electrospray ionization (ESI) operating in negative mode. The analytes were separated by a Waters Xbridge BEH (Ethylene Bridged Hybrid) C₁₈ (25 μm × 2.1 mm × 100 mm) (Milford Massachusetts Stati Uniti) column with an Ultra C₁₈ delay column (5 µm × 30 mm × 2.1 mm, Restek, Centre County, PA, USA), and the sample injection volume was 5 μL. The flow rate was controlled at 0.2 mL/min with a mobile phase of 15 mM ammonium acetate in ultra-pure deionized water (MilliQ) (A) and methanol (B). The overall performances of the analysis have been confirmed from the work cited above [27].

### 2.5. Textural Characterization and Morphology

The surface area Brunauer–Emmett–Teller (BET) multipoint method [28] and textural analysis were carried out via N_2_ adsorption/desorption measurements at the liquid nitrogen temperature (−196 °C) using a Micromeritics 3Flex 3500 analyzer. The sample was pre-treated under vacuum at 350 °C for 2.5 h. The pore distribution was determined by the Barret–Joyner–Halenda (BJH) method [29]. The analysis of micropores was performed by the *t*-test [30], and the total pore volume was determined by the rule of Gurvitsch [31]. Morphology was evaluated by a scanning electron microscope (SEM) analysis using a Zeiss Auriga FESEM without any pre-treatment of the material.

## 3. Results and Discussion

### 3.1. Adsorbent Characterization of Date Biochar

**Figure 2:**
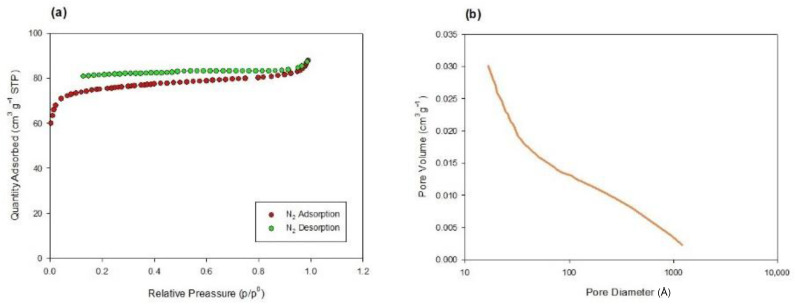
(**a**) N_2_ adsorption/desorption isotherms; (**b**) total pore volume of DS biochar.

**Figure 3:**
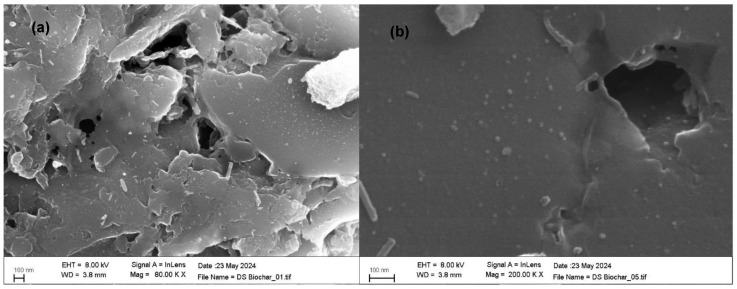
SEM images of DS (**a**,**b**), provided by the CNIS institute.

The PW biochar was characterized in a previous study [26], while the textural characterization and morphology of DS biochar were analyzed in the present investigation. Specific surface area and total pore volume data are reported in Table 1 while the N_2_ adsorption/desorption isotherms are shown in Figure 2a, the pore volume graph is in Figure 2b, and SEM images are in Figure 3. Figure 2a shows a deviation between sorption curves, leading to their lack of meeting. In the literature, this phenomenon is called “open hysteresis” and it is usually present in N_2_ isotherms of biochars deriving from pyrolysis [32]. This behavior has been widely investigated, and it is probably related to (1) pore swelling during adsorption due to the adsorbate (N_2_) penetrating into the pores, thereby causing a deformation and consequentially an expansion of the pore volume and (2) the unreached desorption pressure of the pore blocking the fluid from evaporating so that the adsorbate remains trapped in the cavities [33,34]. Both DS and PW materials show high surface area values, mostly due to micropores, and the great total volumes are principally related to mesopores. However, in the PW biochar, the amount of mesopores is about six times higher than in the DS biochar (Table 1). Moreover, for both PW and DS biochars, the meso- and macropores are continuously distributed in a 20–1000 Å range, mostly in the 20–200 Å range, with a maximum distribution of around 100 Å (Figure 2b). These experimental data may be related to the different matrix origins and production processes of the two biochars, evidencing how such factors may affect the overall pore distribution [35]. In fact, although PW and DSs were prepared in similar temperature conditions (850 °C), their production processes differ (direct gasification and pyrolysis, respectively), resulting in a heterogeneous texture and development of porosity. Regarding morphology, SEM analysis could only partially confirm the adsorption/desorption test: results show pores around 100 Å (Figure 3a), lightly evidenced on the surface of the material (Figure 3a,b), but in-depth information about the micropores distribution was limited by the SEM.

### 3.2. Kinetic Tests

**Figure 4:**
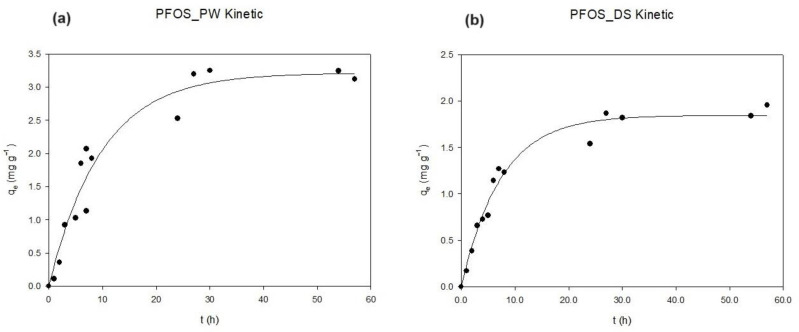
Sorption kinetics of PFOS on PW (**a**) and DS (**b**) at 2 g L^−1^.

Between all the PFASs investigated, the sorption kinetics of PFOS at 1 mg L^−1^ are reported as an example of the adsorbate–adsorbent behavior studied for the two biochars selected (Figure 4). As expected, because of its long chain, log K_ow_ value, and sulfonic group, PFOS interacts effectively with the surface area of both organic materials [36]. The graphs show how the adsorption rate decreases with time until it gradually approaches the equilibrium state. The overall equilibrium state was already reached at 24 h, and the slow adsorption that followed suggests the diffusion of PFOS molecules into the pores of the adsorbents [23].

### 3.3. Isotherm Curves: Pinewood vs. Dates

**Figure 5:**
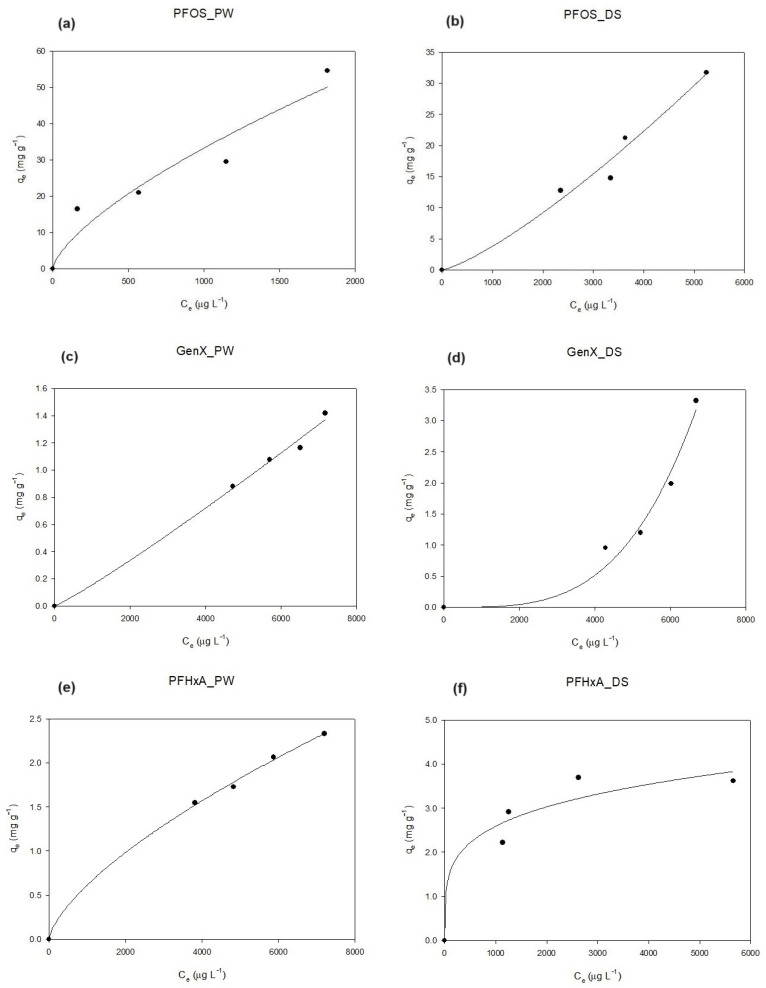

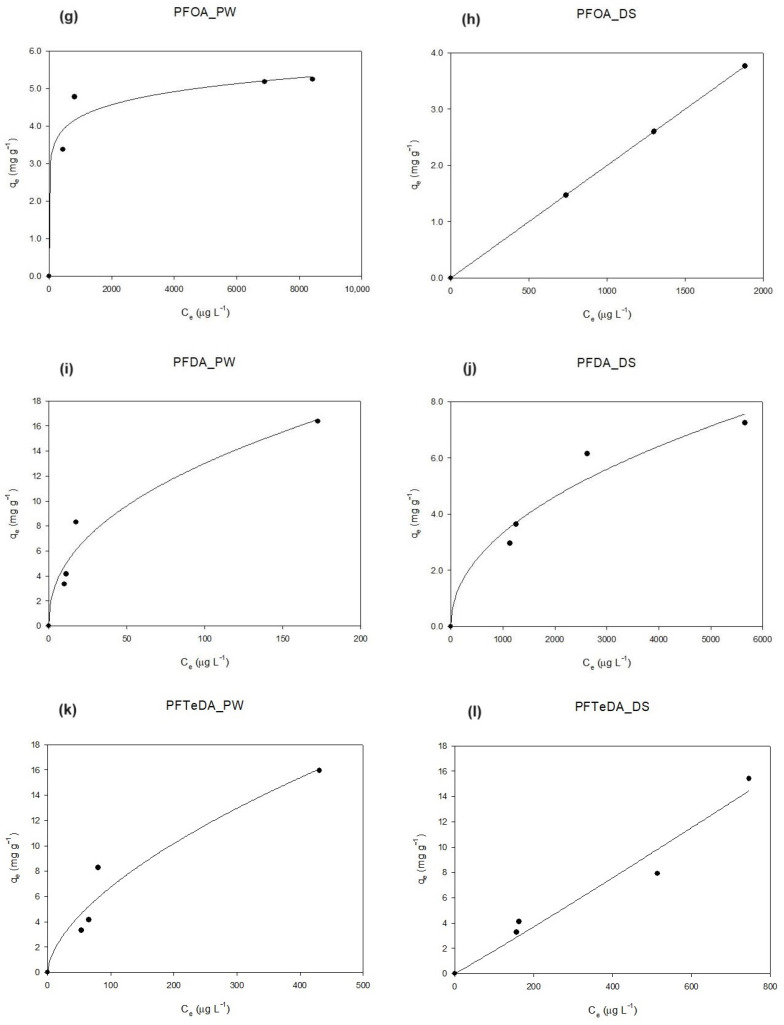
Sorption isotherms of PFOS (**a**,**b**), GenX (**c**,**d**), PFHxA (**e**,**f**), PFOA (**g**,**h**), PFDA (**i**,**j**), and PFTeDA (**k**,**l**) on PW and DS biochar at 10 µg L^−1^.

Sorption isotherms of GenX, PFHxA, PFOA, PFOS, PFDA, and PFTeDA, both on PW and DSs, are reported in Figure 5. The Freundlich model was preferred to Langmuir’s to represent the results because it fits better with the complexity of the adsorption phenomenon investigated with these two heterogenous biochars. Constants and the regression coefficients R^2^ of the Freundlich model are provided in the Supplementary Materials (Table S4). The isotherm curves evidenced three ranges of C_e_ (0–1000, 2000–4000, and 5000–9000 µg L^−1^) related to the type of biochar tested. The highest values are mostly related to DSs, while the intermediate and lowest ones are related to PW. In particular, the majority of C_e_ between 5000 and 9000 µg L^−1^ (Figure 5c–g,j) belong to DSs, while the other two ranges belong to PW (Figure 5a,b,h,i,k,l). Moreover, the PFAS compounds investigated differ in terms of chain length (from 6 to 14 carbon atoms) and functional group (carboxylic and sulfonate). Their chemical structure was shown to play a defining role in the adsorption process, as evidenced by low–intermediate C_e_ values for the long-chain PFASs (PFDA and PFTeDA), implying a major affinity as opposed to the high C_e_ values for the short-chain ones (PFHxA and GenX). As proof of this, PFOS reported the lowest C_e_ values, demonstrating its great affinity with the two carbonaceous materials, probably as a result of the combination of the chain length and sulfonate group (Figure 5a,b) [37,38,39,40]. In fact, PFASs with longer chains are more hydrophobic and their interaction with the carbonaceous adsorbents seems to be stronger and more effective. On the other hand, PFASs with shorter C-F chains show recalcitrant behavior towards both biochars, favoring electrostatic interactions between ions eventually present on the surface of the material [38,39]. PFTeDA (Figure 5k,l), PFOA (Figure 5g,h), and GenX (Figure 5c,d) showed a linear trend in the case of DS isotherms curves, suggesting that the adsorption process occurs in a single layer, probably due to the number of active sites to which the adsorbate can adhere [41]. In the case of PFDA, the same behavior can be observed both with PW and DSs (Figure 5i,j). This could be ascribed to its highly hydrophobic nature for which multilayer sorption is considered favorable, especially at higher equilibrium concentrations [42]. Moreover, while C_e_ values of PFDA and PFOS differ significantly between the two adsorbents, PFTeDA and PFHxA (Figure 5e,f) showed a recurring behavior: C_e_ values are comparable but the isotherms show a different trend, even if only slightly pronounced for the hexanoic compound. The results obtained indicate how the surface area of the two materials investigated, also in terms of pores and chemical composition, affects the overall adsorption of these compounds. This phenomenon has also been studied in the literature, evidencing how the rates of PFAS sorption onto porous materials are closely related to the particle diameter and pore size of sorbents [23,41,42]. In fact, the data show a possible correlation between pore size and adsorption capacity, resulting in lower C_e_ values for PW in the case of long-chain PFASs (PFOS, PFDA, and PFTeDA), in accordance with the mesoporous abundance found by the textural characterization analysis (Table 1). Similar experimental results found in this study have also been reported for AC: the number of CF_2_ units and a functional group of PFASs have been shown to influence its removal efficiency, despite the high surface area of this well-known adsorbent [43,44].

## 4. Conclusions

Sorption tests were carried out to investigate the removal efficiency of six PFASs (PFHxA, GenX, PFOA, PFOS PFDA, and PFTeDA) from aqueous solutions by two biochars derived from organic waste (pinewood and date seeds). The isotherm curves of DSs and PW showed similar trends in relation to PFAS compounds with higher adsorption capacity for the long-chain ones (PFOA, PFOS, PFDA, and PFTeDA). However, peculiar behaviors were also evidenced (PFOS and PFTeDA), probably related to the porous structures and elemental compositions of the two materials. A correlation between pore size and adsorption capacity was also evidenced, showing higher performances for PW. In detail, long-chain PFASs showed low C_e_ values (0–2000 mg L^−1^), especially for PW while intermediate–high C_e_ values (2000–9000 mg L^−1^) were found in relation to short-chain PFASs and DSs. Finally, the adsorption efficiency of the two adsorbents could be summarized as follows: PFOS > PFTeDA > PFDA > PFOA > PFHxA > GenX. These results highlighted an encouraging prospect for the replacement of well-known manufactured adsorbents such as AC in the remediation field. In fact, using alternative biosorbents has less of an environmental and cost-effective impact in the long term. Following this point of view, in order to reach and possibly exceed AC’s performances, further investigation should be explored (i) via column tests (both for single-PFAS compounds and mixtures) and (ii) by studying functionalization procedures to enhance adsorption capacity for both DSs and PW.

## Figures and Tables

**Table 1:** Specific surface area and pore volume of DS and PW biochar.

**Pinewood**		
	**Surface Area (m^2^ g^−1^)**	**Volume Pore (m^3^ g^−1^)**
Total	343 ± 2	0.383
Micropores	224	0.136
Mesopores	119	0.247
**Date Seeds**		
	**Surface Area (m^2^ g^−1^)**	**Volume Pore (m^3^ g^−1^)**
Total	290 ± 4	0.136
Micropores	270	0.110
Mesopores	20	0.026

## Data Availability

The raw data supporting the conclusions of this article will be made available by the authors on request.

## Supplementary Materials

The following supporting information can be downloaded at https://www.mdpi.com/article/10.3390/ma17174169/s1, Table S1: PFAS compounds investigated, relative molecular formulas, weights, and octanol–water partition coefficients (Log K_ow_); Table S2: Electronic parameters and retention time (RT) of each PFAS compound investigated; Table S3: Elution gradient in column for PFAS analysis; Table S4: Isotherm parameters, with corresponding regression coefficients (R^2^).
