# Supplementary material for: Adsorption Technology for PFAS Removal in Water: Comparison between Novel Carbonaceous Materials
Source: Materials (Basel). 2024 Aug 23;17(17):4169. doi: 10.3390/ma17174169 (PMC11395723; doi:10.3390/ma17174169)
Supplement: Supplementary file 1 [file materials-17-04169-s001.zip › materials-3115412-supplementary.pdf]

Supplementary Materials

Table S1. PFAS compounds investigated. relative molecular formulas. weights and octanol-water partition coefficients (Log K_{ow})

Name	Acronymous	Molecular Formula	Molecular Weight (g mol^{-1})	Carbon Number	Log K_{ow}	Manufactoring Company
Perfluorohexanoic acid	PFHxA	$C_5F_{11}COOH$	314.1	6	4.06	LGC Standards Ltd (Milan. Italy)
Perfluoro-n-[$^{13}C_2$]hexanoic acid	m-PFHxA	$[^{13}C]_2C_4F_{11}COOH$	316.1	6		
Ammonium perfluoro(2-methyl-3-oxahexanoate)	GenX	$C_6H_4F_{11}NO_3$	347.1	6	3.6	
Perfluorooctanoic acid	PFOA	$C_7F_{15}COOH$	414.1	8	5.3	
Perfluorooctanesulfonic acid	PFOS	$C_8F_{17}SO_3H$	500.1	8	6.3	
Sodium perfluoro-1-[$^{13}C_4$]octanesulfonate	m-PFOS	$[^{13}C]_4$ $C_4F_{17}SO_3H$	504.1	8		
Perfluorodecanoic acid	PFDA	$C_9F_{19}COOH$	514.1	10	6.5	
Perfluorotetradecanoic acid	PFTeDA	$C_{13}F_{27}COOH$	714.1	14	7.19	

Table S2. Electronic parameters and retention time (RT) of each PFAS compound investigated.

Compound	Molecular weight (g mol^{-1})	Precursor ion (m/z)	Product ion (m/z)	DP (V)	Ce (V)	Rt (min)
PFHxA	314	313	119	-10	-38	6.89
m-PFHxA	314	315	119	-10	-38	6.89
GenX	284	285	184.6	-60	-25	7.74
PFOA	412	413	369	-10	-20	12.31
PFOS	500	499	79.8	-117	-120	13.61
m-PFOS	504	503	79.8	-117	-120	13.61
PFDA	514	513	169	-25	-16	14.12
PFTeDA	713	712.8	668.6	-10	-35	19.53

Table S3. Eluition gradient in column for PFAS analysis.

Time (min)	Phase A (H_2O+15Mm CH_3COONH_4) (%)	Phase B (MeOH) (%)
0	50	50
1	10	90
22	10	90
23	0	100

Table S4. Isotherm parameters (K_F, n) of both PW and DS adsorption tests for each PFAS compound, with corresponding regression coefficients (R^2).

	K_F (L g^{-1})	n	R^2
PFDA DS	1.26E-01	4.74E-01	0.98
PFDAPW	1.75	4.36E-01	0.97
PFOA DS	2.00E-03	1.00	1.00
PFOA PW	2.05	1.05E-01	0.98
GenX DS	8.17E-14	3.55	0.99
GenX PW	7.72E-05	1.10	0.99
PFHxA DS	5.43E-01	2.26E-01	0.97
PFHxA PW	5.80E-03	6.76E-01	0.99
PFOS DS	5.00E-04	1.28	0.99
PFOS PW	2.89E-01	6.87E-01	0.96
PFTeDA DS	1.52E-02	1.04	0.98
PFTeDA PW	4.34E-01	5.96E-01	0.97
